# Precise analysis of single small extracellular vesicles using flow cytometry

**DOI:** 10.1038/s41598-024-57974-3

**Published:** 2024-03-29

**Authors:** Hisano Kobayashi, Takayuki Shiba, Takeshi Yoshida, Dilireba Bolidong, Koroku Kato, Yoshiki Sato, Mao Mochizuki, Takafumi Seto, Shuichi Kawashiri, Rikinari Hanayama

**Affiliations:** 1 Department of Immunology, Graduate School of Medical Sciences, Kanazawa University, Kanazawa, Ishikawa Japan; 2 Oral and Maxillofacial Surgery, Graduate School of Medical Sciences, Kanazawa University, Kanazawa, Ishikawa Japan; 3 WPI Nano Life Science Institute (NanoLSI), Kanazawa University, Kanazawa, Ishikawa Japan; 4 Institute of Science and Engineering, Faculty of Frontier Engineering, Kanazawa University, Kanazawa, Ishikawa Japan; 5 Meiwafosis Co. LTD, Tokyo, Japan

**Keywords:** Flow cytometry, ESCRT

## Abstract

Methods that enable specific and sensitive quantification of small extracellular vesicles (sEVs) using flow cytometry are still under development. Aggregation or adsorption of antibodies causes sub-nano sized particles or non-specific binding and largely affects the results of flow cytometric analysis of single sEVs. Comparison of control IgG and target-specific IgG is inappropriate because they have different characters. Here, we evaluate four preparation methods for flow cytometry, including ultracentrifugation, density gradient centrifugation, size exclusion chromatography (SEC), and the TIM4-affinity method by using tetraspanin-deficient sEVs. The ultracentrifugation or density gradient centrifugation preparation method has large false-positive rates for tetraspanin staining. Conversely, preparation methods using SEC or the TIM4-affinity method show specific detection of single sEVs, which elucidate the roles of sEV biogenesis regulators in the generation of sEV subpopulations. The methods are also useful for the detection of rare disease-related markers, such as PD-L1. Flow cytometric analysis using SEC or the TIM4-affinity method could accelerate research into sEV biogenesis and the development of sEV-based diagnostics and therapies.

## Introduction

Most cells in the human body secrete extracellular vesicles (EVs), including exosomes, microvesicles, and apoptotic bodies^1^. Exosomes are derived from multivesicular endosomes (MVEs) that develop through various pathways, such as the ESCRT^2^, syntenin-syndecan^3^, and ceramide pathways^4^, and are secreted by the fusion of MVEs with plasma membranes. Microvesicles and apoptotic bodies are secreted as outward-budding plasma membranes. Different pathways of biogenesis and types of EVs correspond to different cargoes, including proteins, nucleic acids, and lipids, resulting in EV heterogeneity^5^. However, the heterogeneity of EVs is not yet fully understood because single-particle analysis methods are not readily available.

Small EVs (sEVs, 30–150 nm diameter) are involved in the progression of various diseases, such as cancer and neurodegenerative diseases^6^. As their involvement in diseases becomes clearer, sEVs are thought to be promising targets for the diagnosis of sEV-related diseases^6^. However, because most sEVs in body fluids are secreted from healthy cells, a highly sensitive detection system for single sEVs and identification of highly specific markers is critical for accurate diagnosis. Conventional sEV-based diagnostic methods use bulk sEVs, which limits the detection of small amounts of sEVs from diseased cells. Conversely, single sEV analysis is more suitable for early diagnosis because the signal per particle is not weakened.

Recent advances in flow cytometry have allowed high-throughput and multiple-color analyses of sEVs^7–9^. To specifically analyze sEVs by antibody staining, it is important that unbound antibodies be completely removed by washing, because aggregated antibodies can be detected as nonspecific signals. Comparison of control IgG and target-specific IgG is inappropriate to prove the validity of the method because they have different aggregation and adsorption properties. Comparing wild-type and target molecule-deficient sEVs seems essential for developing a precise analysis of single sEVs using flow cytometry, but it has rarely been adopted. Methods of sEV purification, such as polymer precipitation, ultracentrifugation^10^, density gradient centrifugation^11^, size exclusion chromatography (SEC)^12^, and affinity isolation methods^10,13^ are used to remove unbound antibodies; however, optimal methods and contexts have not yet been elucidated. As polymer precipitation isolates not only sEVs but also many proteins, including unbound antibodies, they cannot be used for antibody staining. Therefore, we compared the performance of ultracentrifugation, density gradient centrifugation, SEC, and affinity isolation methods in removing unbound antibodies. SEC separates sEV from proteins and other impurities using a column packed with porous beads with pore sizes of 35–70 nm. In this study, an ability of SEC columns with a pore size of 35 nm to remove unbound antibodies was evaluated. Magnetic beads conjugated with antibodies against tetraspanins are often used for affinity isolation methods, but it is very difficult to release sEVs from antibody-conjugated beads, and only limited subpopulations of sEVs can be isolated. To resolve the problem, we developed a TIM4-affinity method, which captures bulk sEVs by recognizing phosphatidylserine (PS) on sEV membranes and releases captured sEVs as single particles by adding ethylene diamine tetra acetic acid (EDTA)^13^.

## Materials and methods

### Cells and plasmids

Human embryonic kidney cell line 293T (Riken BRC, Ibaraki, Japan), human head and neck squamous cell carcinoma cell lines, OSC-19 cells (#0198, Japanese Cancer Research Resources Bank [JCRB], Osaka, Japan), OSC-20 cells (#0197, JCRB), and HOC313 cells^14^ (kindly gifted by Dr. M. Nagayama) were maintained in Dulbecco’s modified Eagle medium (DMEM) supplemented with 100 units/mL penicillin, 100 µg/mL streptomycin, 2 mM l-glutamine, and 10% heat-inactivated fetal bovine serum (FBS) (Nichirei Biosciences, Tokyo, Japan). All cells were cultured at 37 °C with 5% CO_2_ and routinely tested for mycoplasma using PCR targeting the 16S ribosome.

CD9, CD63, CD81, ALIX, HGS, and TSG101 knockout (KO) 293T cells were established using the CRISPR/Cas9 system. The pX330-Puro plasmid^15^ targeting the sequence CCAGCGGGAAACGCTGAAAGC at human *CD9* exon 5, CCTTCCATGTCGAAGAACCGA at human *CD63* exon 6, GGGCTGCTACGGGGCCATCCAGG at human *CD81* exon 3, CGTCCGCTGGACAAGCACGAGGG at human *PDCD6IP* (also known as *ALIX)* exon 1, CCCACACGTCGCCTTGTATGCCC at human *HGS* exon 3, or CCTACTAGTTCAATGACTATTA at human *TSG101* exon 4 (underlined sections represent a PAM sequence) was transfected into 293T cells using FuGENE6 (Promega, Madison, WI, USA). For the selection of ALIX, HGS, or TSG101 KO cells, the cells were cultured in medium containing 1 µg/mL puromycin for 3 d from 2 d after transfection. The expression of each protein was confirmed by western blot analysis. For the selection of CD9, CD63, and CD81 KO cells, the cells were stained with human CD9, CD63, or CD81 antibodies, and the negative population was sorted using a cell sorter SH800S (SONY, Tokyo, Japan). The antibodies used in this study are listed in Supplementary Table 1.

The pCDNA3.1-hCD63EGFP plasmid encoding the fusion protein human CD63 and EGFP under the control of the CMV promoter was cloned as follows: The Human *CD63* DNA fragment was amplified from K562 cDNA by PCR using PrimeSTAR (Takara Bio, Shiga, Japan) and paired primers (forward: 5′-CTAGCTAGCCATGGCGGTGGAAGGAGG-3′; reverse: 5′-AGCTACCGGTCTCATCACCTCGTAGCCACT-3′), and inserted into the *Nhe*I/*Age*I site of the pLJM1-EGFP plasmid (Cat. # 19319; Addgene, Watertown, MA, USA) to generate the pLJM1-hCD63EGFP plasmid. The fusion genes human *CD63* and *EGFP* were amplified from pLJM1-hCD63EGFP using paired primers (forward: 5′-CCGGAATTCGCTAGCCATGGCGGTGGAAGGA-3′; reverse: 5′-CCGGGTACCACTTGTACAGCTCGTCCATGCC-3′) and inserted into the *Eco*RI/*Kpn*I site of pcDNA3.1 (–) (Thermo Fisher Scientific, Waltham, MA, USA) to generate the pcDNA3.1-hCD63EGFP plasmid.

### Preparation of cultured supernatant for sEV analysis

To prepare sEV-depleted FBS, 10 mL of FBS was mixed with 2 mL of 50% PEG-10,000 (Merck, Darmstadt, Germany), gently rotated overnight at 4 °C, and then the mixture was centrifuged at 1500 × *g* at 4 °C for 30 min. The supernatant was filtered through a 0.22 µm polyvinylidene difluoride (PVDF) filter (Merck) and added to 500 mL of Advanced DMEM (Thermo Fisher Scientific) supplemented with 100 units/mL penicillin, 100 µg/mL streptomycin, and 2 mM l-glutamine. The cells were cultured in Advanced DMEM with 2% sEV-depleted FBS until they reached ~ 80% confluency. The cultured supernatant was harvested and sequentially centrifuged at 300 × *g* for 5 min, 2000 × *g* for 20 min, and 10,000 × *g* for 30 min at 4 °C to collect the supernatant of centrifugation at 10,000 × *g* (10 K sup). The sEVs contained in 7 mL of the 10 K sup were concentrated by ultracentrifugation at 100,000 × *g* for 2 h at 4 °C using a CS100FNX with an S50ST swing rotor (Eppendorf Himac Technologies, Ibaraki, Japan) and the pellet was resuspended in 70 µL of immobilizing/washing buffer with binding enhancer to generate UC sEVs. The 10 K sup or UC sEVs were used for staining immediately or after storage at 4 °C up to 5 days.

To prepare sEVs expressing human CD63EGFP or mock sEVs, the pcDNA3.1-hCD63EGFP plasmid or pcDNA3.1 (–) plasmid was transfected into 293T cells using PEI Max (Polysciences, Niles, IL, USA). The culture medium was changed to DMEM with 10% FBS 5 h after transfection, and Advanced DMEM with 2% sEV-depleted FBS 24 h after transfection. After an additional 48 h of cell culture, the 10 K sup or UC sEVs were recovered from the culture medium using the methods described above. To analyze PS^+^- and PS^–^-sEVs, they were separated from the UC sEVs of 293T cells using a MagCapture Exosome Isolation Kit PS Ver.2 (FUJIFILM Wako Pure Chemical, Osaka, Japan) according to the manufacturer’s instructions. Briefly, TIM4-beads were prepared by mixing 3 mg of streptavidin magnetic beads with 5 µg of TIM4-biotin. We applied 1 mL of 1 × 10^11^ sEVs/mL UC sEVs (whole sEVs) to 3 mg TIM4-beads with rotation at 4 °C for 1 h. The supernatant was collected and reacted with TIM4-beads five times to completely remove PS^+^-sEVs and isolate PS^–^-sEVs. The PS^+^-sEVs bound to the TIM4-beads were washed with immobilizing/washing buffer with a binding enhancer three times and eluted from the beads by adding 50 µL of elution buffer twice to isolate PS^+^-sEVs. To analyze PD-L1^+^-sEVs, the cells were cultured in Advanced DMEM with 2% sEV-depleted FBS containing 0 or 100 ng/mL recombinant human IFN-γ (Cat. # 570202; BioLegend, San Diego, CA, USA) at 37 °C for 24 h. The 10 K sup was recovered from the culture medium using the method described above. Characterization of sEVs was performed according to the MISEV2023 guidelines^16^.

### Staining sEVs and washing by ultracentrifugation

UC sEVs (1.0 × 10^9^ sEVs) were stained with fluorescence-conjugated antibodies in 20 µL immobilizing buffer at room temperature for 2 h. The stained sEVs were washed in 7 mL PBS(–) by ultracentrifugation at 100,000 × *g* for 2 h using the CS100FNX with the S50ST swing rotor. After discarding the supernatant, the pellet was suspended in 100 µL PBS(–) with 1% EV-Save (FUJIFILM Wako Pure Chemical). The antibodies used for staining sEVs are listed in Supplementary Table 1.

### Staining sEVs and washing by density gradient centrifugation

UC sEVs (1.0 × 10^9^ sEVs) were stained with fluorescence-conjugated antibodies in 20 µL immobilizing buffer at room temperature for 2 h. Gradient density fractions were prepared in a tube for ultracentrifugation with 1 mL of 0, 10, 20, 30, 40, and 50% iodixanol solution (OptiPrep, Axis-Shield Diagnostics, Luna Place, Scotland). After adding 980 µL PBS(–) to the stained sEVs, the mixture was placed at the top of the gradient density fractions and ultracentrifuged at 100,000 × *g* for 16 h using the CS100FNX with the S50ST swing rotor. Each fraction (1 mL) was carefully collected in a microtube, and the density was determined by measuring the absorbance at 340 nm. The sEV-containing fraction (1.08 g/mL density) was transferred into a dialysis membrane with 100 kDa MWCO and rotated overnight in PBS(–). The sEVs were collected from the membrane, and EV-Save was added at 1%.

### Staining sEVs and washing by SEC

UC sEVs (1.0 × 10^9^ sEVs) were stained with fluorescence-conjugated antibodies in 20 µL immobilizing buffer at room temperature for 2 h. After equilibrating a column of SEC with a pore size of 35 nm (qEV35, IZON, Christchurch, New Zealand) with 8.5 mL of PBS(–) buffer, 20 µL stained sEVs and 480 µL PBS(–) was applied to the column. After washing the column with PBS(–), the sEVs were eluted with PBS(–), and 20 µL EV-Save was added.

### Staining sEVs and washing by TIM4-affinity method

TIM4-beads were prepared by mixing 0.06 mg of streptavidin magnetic beads with 0.1 µg of TIM4-biotin contained in the MagCapture Exosome Isolation Kit PS Ver.2 (FUJIFILM Wako Pure Chemical). The sEVs were stained before or after being captured by TIM4-beads. In case of staining prior to capture, the UC sEVs (1.0 × 10^9^ sEVs) were stained with fluorescence-conjugated antibodies in 20 µL immobilizing buffer for 2 h. After adding 980 µL of immobilizing buffer with a binding enhancer to the stained sEVs, they were reacted with 0.06 mg of TIM4-beads with rotation at 4 °C for 1 h. In the case of staining after capture, the sEVs contained in 1 mL of the 10 K sup were captured using TIM4-beads with rotation at 4 °C for 1 h and washed with 1 mL TBS-Ca. The sEVs captured using TIM4-beads were stained with fluorescence-conjugated antibodies in 20 µL immobilizing buffer with a binding enhancer at room temperature for 2 h. After washing the beads with TBS-Ca three times, sEVs were eluted from the TIM4-beads by incubating the beads for 10 min in 50 µL elution buffer [2 mM EDTA/PBS(-)] with 1% EV-Save twice.

### Flow cytometry analysis for sEVs

Milli-Q water was filtered through a Millex-GS 0.22 µm MCE membrane (Merck) three times for use of the sheath flow of sEV flow cytometry. The sEVs were analyzed using a Flow Nanoanalyzer (NanoFCM, Xiamen, China) equipped with two lasers (488 nm and 638 nm) and three band-pass filters (488/10, 525/40, and 580/40). All measurements were performed according to the manufacturer’s protocol. Briefly, silica nanoparticle cocktail (68–155 nm, Cat. # S16M-Exo, NanoFCM) and polystyrene 250 nm beads (QC Beads, Cat. # S08210, NanoFCM) were used as the size and particle concentration references, respectively. PBS(–) was measured to determine a threshold for the SS-H channel, which was set to “mean + 3 × SD” of SS-H in PBS(–). sEVs were diluted with PBS(–) at a concentration of 1.0 × 10^8–9^ sEVs/mL to run sEVs at a flow rate of 50–200 events per second and more than 3000 events were recorded during 60 s of measurement. All samples were run under identical pressure (1 kPa), and the signals of SS, FITC, and PE were recorded. Flow cytometry analysis of sEVs was performed following the MISEV2023 guidelines^16^ and the MIFlowCyt-EV2020 guidelines^17^. The detailed information is provided in Supplementary Information 1.

### Nanoparticle tracking analysis (NTA)

The particle concentration of sEVs was determined using a NanoSIGHT NS300 equipped with 405 nm laser (Malvern Panalytical, Malvern, United Kingdom). sEVs were diluted 10- to 1000-times with PBS(–) to record 10–50 particles/frame. The movement of sEVs was recorded three times at camera level 15, slider shutter 1206, slider gain 245, room temperature for 30 s and analyzed using NTA3.1 software (Malvern Panalytical) at detect threshold 5.

### Imaging sEVs under atomic force microscopy (AFM)

The sEVs isolated by each method were observed under AFM by following previously reported methods^18,19^. Briefly, 50 μL of 0.01% Poly-L-lysine (PLL) solution (Merck) was coated on a mica substrate under a clean desiccator for about 5 min. After rinsing the mica with 100 μL of Milli-Q water 10 times, 100 μL of each sEVs (1 × 10^10^ EVs/mL) were immobilized onto the PLL-coated mica substrate for 15 min at room temperature. The sEVs were rinsed with Milli-Q water several times and then were placed in PBS(–) to observe them in liquid state under Bruker BioScope Resolve AFM system (Bruker, Billerica, MA) equipped with a 240AC-NG cantilever (OPUS, Sofia, Bulgaria) with a nominal spring constant of 200 N/m and a nominal tip radius of 8 nm. Scanning was performed in liquids at room temperature using PeakForce Tapping® mode with ScanAsyst, tapping amplitude 50 nm, and tapping frequency 1 kHz. Scan size was 2 × 2 μm^2^ with a minimum pixel resolution of 512 pixels per line. The AFM image processing was performed using the NanoScope Analysis software (version 1.9, Bruker).

### Flow cytometry analysis of cells and sorting

Cells were detached from the dishes by treatment with accutase (FUJIFILM Wako Pure Chemical) for 3 min. After removing the accutase by centrifugation at 300 × *g* for 3 min, the cells were suspended in PBS( −) containing 2% FBS. Approximately 1 × 10^6^ cells were stained with an antibody in 100 µL PBS( −) containing 2% FBS at 4 °C for 60 min. After washing the cells twice with PBS(–) containing 2% FBS, they were suspended in 500 µL PBS(–) containing 2% FBS. Cellular analysis was performed using a flow cytometer SA3800 (SONY). Cell sorting was performed using a cell sorter SH800S (SONY) equipped with a 100-μm microfluidic sorting chip at ultra purity sorting mode. The antibodies used for cellular staining are listed in Supplementary Table 1.

### Data analysis

Flow Cytometry Standard (FCS) files with FCS 3.1 format were analyzed using FlowJo (v10.8, BD Biosciences, Franklin Lakes, NJ). The threshold of SS-H was set using PBS(−), blank sample. The SS-H, FITC-A, and PE-A signals of all sEVs were analyzed. The size of sEVs was calculated based on the SS-H signals of standard size beads.

## Results

### Highly pure washing methods are necessary for detecting single sEVs in flow cytometry analysis

**Figure 1:**
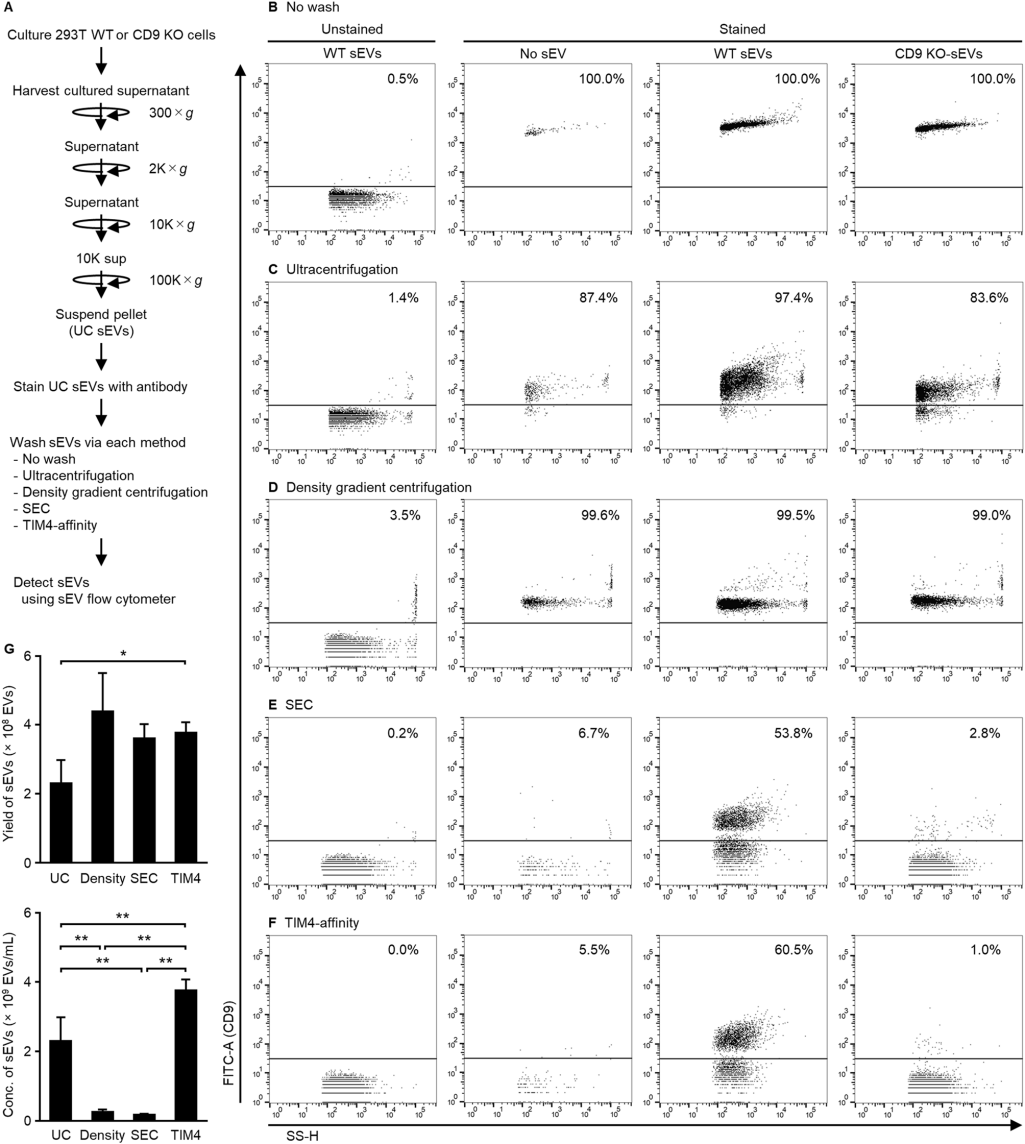
Evaluation of different washing methods for sEVs stained with a single antibody. (**A**) The overview of sEV preparation by different washing methods for flow cytometry analysis. 293T WT or CD9 KO cells were cultured in Advanced DMEM-2% sEV-depleted FBS for 48 h. After removing cells and cellular debris, the cultured supernatant was concentrated via ultracentrifugation to generate UC sEVs. The UC sEVs were stained with FITC-anti human CD9 antibody for 2 h. The stained sEVs were not washed (**B**) or washed via ultracentrifugation (**C**), density gradient centrifugation (**D**), SEC using qEV35 (**E**), or the TIM4-affinity method (**F**). Side scatter (SS) and FITC intensity of the sEVs were detected using NanoFCM. (**G**) Yield or concentration of sEVs isolated by each method was measured by NTA. Data show mean ± SD. *P < 0.05, **P < 0.01, paired *t* test with Bonferroni’s correction, *n* = 4. Sizes of the sEVs are presented in Supplementary Fig. 2.

**Figure 2:**
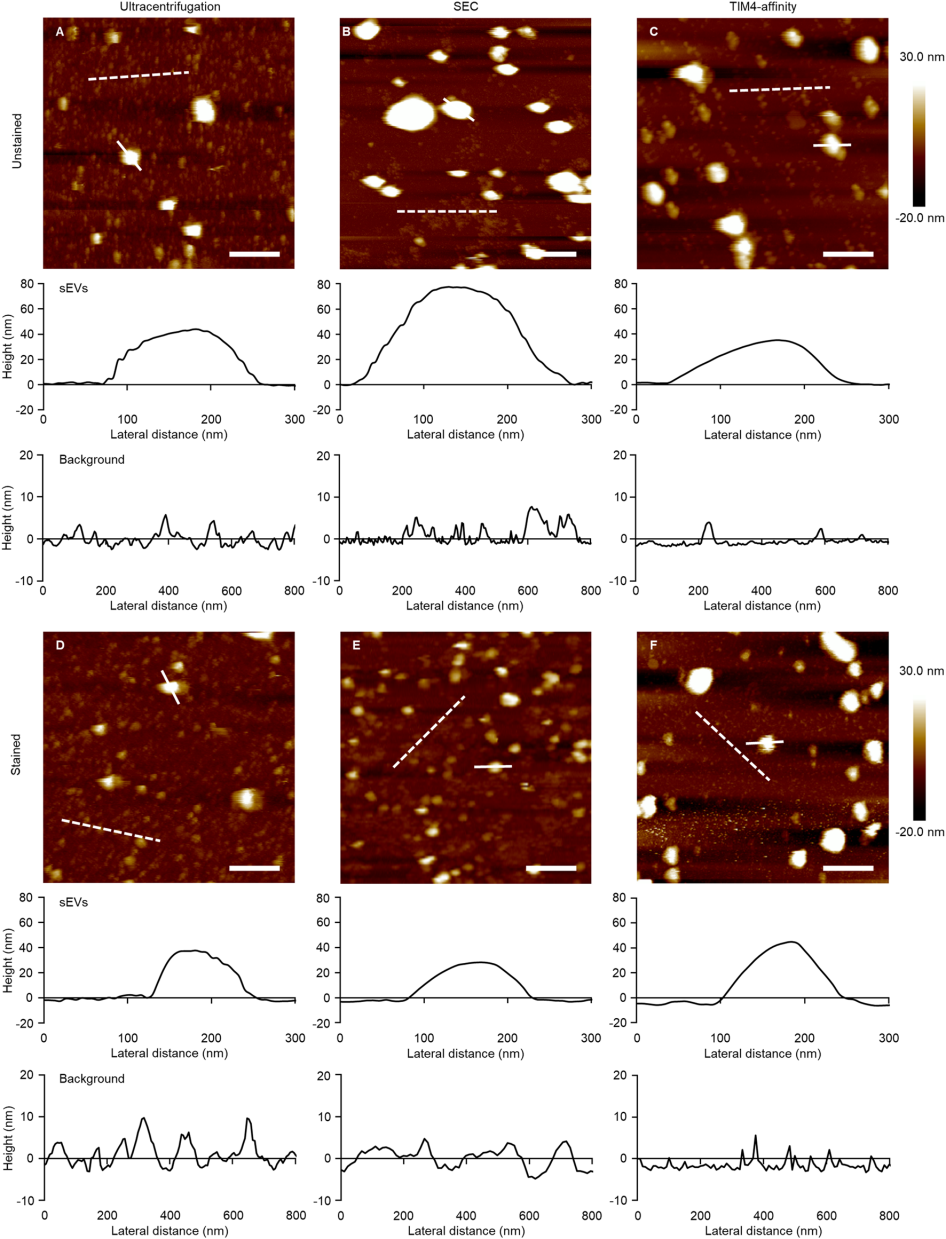
AFM observation of sEVs washed by different methods. UC sEVs from 293T WT cells were stained with FITC-anti human CD9 antibody for 2 h. The stained sEVs were washed via ultracentrifugation (**A**: unstained, **D**: stained), SEC using qEV35 (**B**: unstained, **E**: stained), or TIM4-affinity method (**C**: unstained, **F**: stained). The sEVs were observed under an AFM, the scale bar represents 400 nm; a color tone shows height. A cross-section of a typical sEV and a typical background area with no sEV, indicated by a solid line and a dashed line respectively, is shown in the lower panel of **A**-**F**.

The purity of sEVs is one of the most important factors in their detection. We validated the purification requirement for sEV flow cytometry analysis by detecting sEVs expressing fluorescent proteins. A plasmid expressing CD63EGFP or mock plasmid was transfected into 293T cells, and the sEVs recovered from the cultured supernatant by ultracentrifugation were analyzed by sEV flow cytometry. Analysis of mock sEVs isolated by ultracentrifugation showed that most sEVs were 40–80 nm in diameter (Supplementary Fig. 1). Analysis of CD63EGFP-sEVs isolated by ultracentrifugation showed that 69.5% of sEVs were EGFP^+^-sEVs. Ultracentrifugation can be used to detect sEVs using an sEV flow cytometer. Subsequently, we validated the requirement for a washing step after staining the sEVs with antibodies. The sEVs from wild-type (WT) cells or CD9 KO cells were stained with FITC-anti CD9 antibody and washed using different methods, including ultracentrifugation, density gradient centrifugation, SEC, or the TIM4-affinity method (Fig. 1A). In the absence of washing, both WT-sEVs and CD9 KO-sEVs were detected as CD9-positive (Fig. 1B). Additionally, sEV flow cytometry detected frequent FITC-positive signals corresponding to 50–80 nm particle size in a sample containing FITC-anti CD9 antibody and no sEV (Supplementary Fig. 2), suggesting that some antibodies formed aggregates and were detected by sEV flow cytometry. Therefore, flow cytometry analysis of sEVs requires an appropriate washing step to remove antibodies. Washing via ultracentrifugation resulted in a CD9 KO-sEV detection rate of 83.6% (Fig. 1C), indicating that the removal of antibodies was inefficient. In addition, observation under AFM revealed that the sample via ultracentrifugation contained sEVs of 60–100 nm in diameter and many protein-aggregated particles (Fig. 2A). The protein-aggregated particles of over 5 nm in height increased by the antibody staining (Fig. 2D). Washing via density gradient centrifugation resulted in many positive signals in a sample containing FITC-anti CD9 antibody and no sEV (Fig. 1D) and was unable to separate antibodies from sEVs. Western blot analysis of each density fraction revealed that the sEVs and the antibody were distributed in the range of 1.04–1.15 g/mL and 1.04–1.08 g/mL, respectively (Supplementary Fig. 3). Washing via SEC resulted in 50–70 nm in size, 53.8% positivity for WT-sEVs and 2.8% positivity for CD9 KO-sEVs (Supplementary Fig. 2 and Fig. 1E). which was highly effective in comparison to the previous washing methods. Compared to the ultracentrifugation and TIM4-affinity methods, SEC recovered sEVs at a lower concentration (1.74 × 10^8^ sEVs/mL) but with a comparable total yield (Fig. 1G), which was sufficient for measurement by sEV flow cytometry. AFM showed that protein aggregates were considerably washed away (Fig. 2B), whereas aggregated antibodies of around 5 nm in height were present (Fig. 2E). Washing with the TIM4-affinity method resulted in 60.5% positivity for WT-sEVs and 1.0% positivity for CD9 KO-sEVs (Fig. 1F), showing results similar to that of SEC. The TIM4-affinity consistently provided a high concentration of sEVs (3.77 × 10^9^ sEVs/mL, Fig. 1G). Further, AFM revealed that protein aggregates were clearly removed using the TIM4-method (Fig. 2C, F). The TIM4-affinity also showed good performance in staining for CD63 and CD81 (Supplementary Figs. 4 and 5). Staining with different concentrations of antibody showed that CD9 staining was insufficient at concentrations ≤ 2.5 µg/mL and sufficient at ≥ 5 µg/mL (Supplementary Fig. 6B). Even after treatment with 10 µg/mL antibody, the antibody was completely washed away and nonspecific binding to CD9 KO-sEVs was not observed (Supplementary Figs. 6A and C). These data indicate that highly pure isolation methods, including SEC and the TIM4-affinity method, are appropriate for staining sEVs with antibodies and the antibody concentration is an important factor.

**Figure 3:**
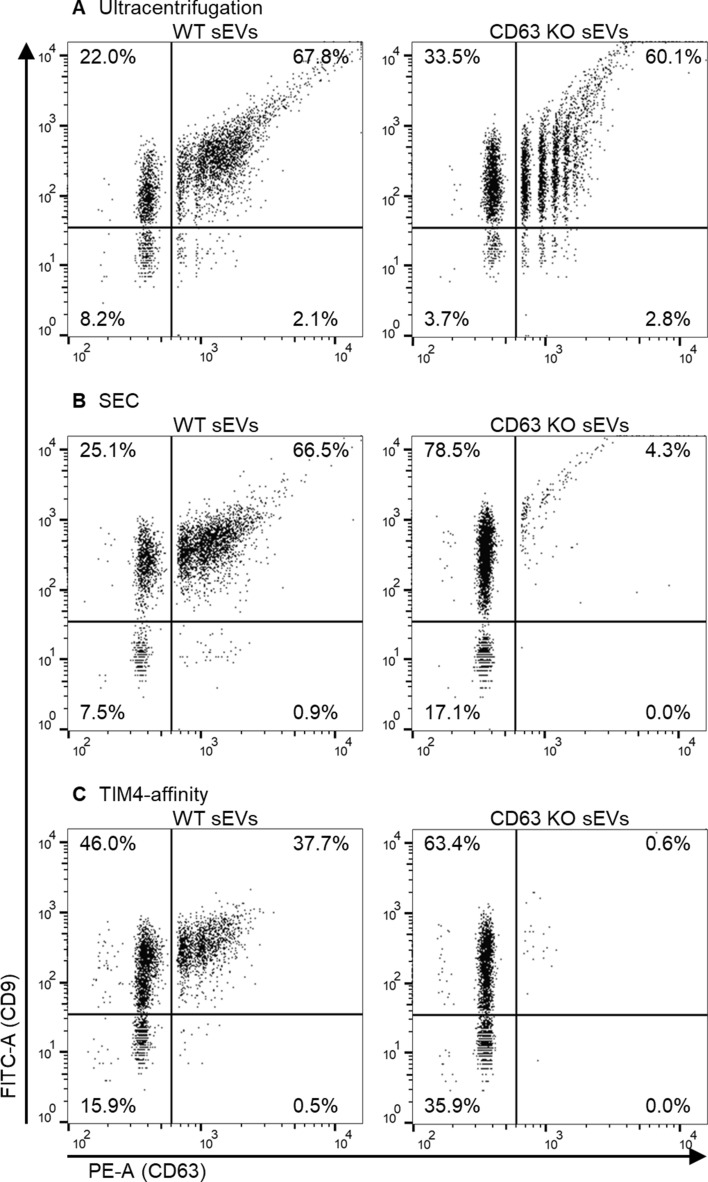
Evaluation of different washing methods to analyze sEV heterogeneity. UC sEVs from 293T WT or CD63 KO cells were stained with FITC-anti CD9 antibody and PE-anti CD63 antibody for 2 h. The sEVs were washed via ultracentrifugation (**A**), SEC using qEV35 (**B**), or the TIM4-affinity method (**C**). SS, FITC, and PE intensity of the sEVs were detected using NanoFCM.

Analysis of sEV heterogeneity is useful for understanding the mechanisms of sEV biogenesis and sEV-related diseases, but analysis of sEVs at the single-particle level is not commonly applied because single particles are difficult to detect, stain with multiple antibodies, or separate from background noise/signals. Accordingly, we confirmed whether highly pure isolation methods could be applied to detect double-stained sEVs. The sEVs from 293T WT or CD63 KO cells were double-stained with FITC-anti CD9 and PE-anti CD63 antibodies and washed with ultracentrifugation, SEC, or the TIM4-affinity method. Prior to the experiments, we confirmed that the single-staining signals of each antibody rarely leaked into the other fluorescent channels (Supplementary Fig. 7). Using ultracentrifugation, more than 60% of CD63 KO-sEVs were detected as CD63^+^-sEVs, indicating that washing with ultracentrifugation was ineffective (Fig. 3A). In SEC with qEV35, 4.3% of CD63 KO-sEVs and 66.5% of WT-sEVs were detected as CD63^+^-sEVs (Fig. 3B). With the TIM4-affinity method, 0.6% of CD63 KO-sEVs and 37.7% of WT-sEVs were detected as CD63^+^-sEVs (Fig. 3C). Furthermore, the rates of CD9^+^-sEVs were similar between WT-sEVs and CD63 KO-sEVs in both the SEC and TIM4-affinity methods, indicating that double staining does not promote competition between antibodies (Fig. 3B, C).

**Figure 4:**
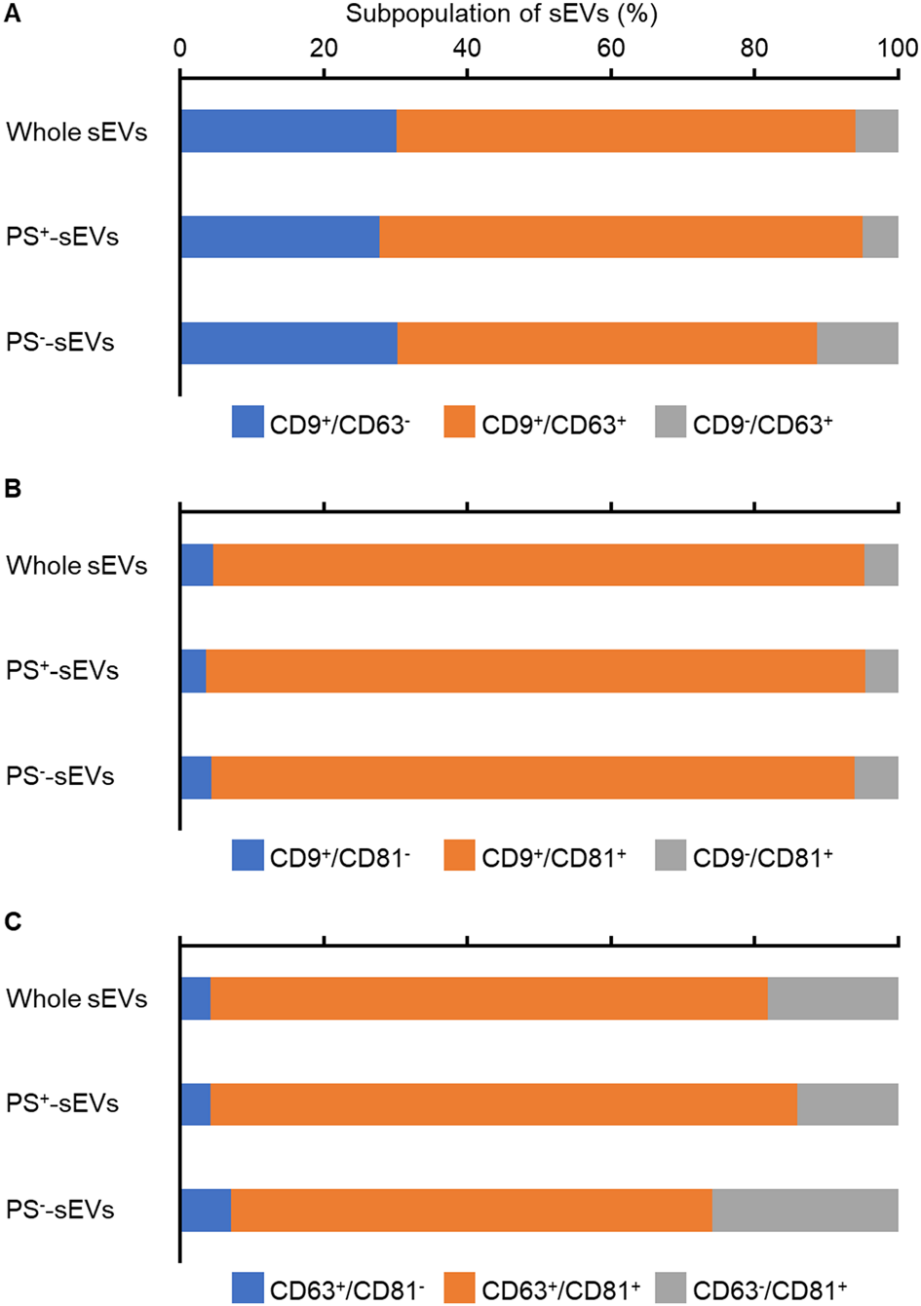
Analysis of sEV heterogeneity between PS^+^-sEVs and PS^–^-sEVs. 293T cells were cultured in Advanced DMEM-2% sEV-depleted FBS for 48 h and sEVs were recovered by ultracentrifugation. Whole sEVs were separated into PS^+^-sEVs and PS^–^-sEVs using the TIM4-affinity isolation method. Whole sEVs, PS^+^-sEVs, or PS^–^-sEVs were stained with FITC-anti CD9 and PE-anti CD63 antibodies (**A**), FITC-anti CD9 and PE-anti CD81 antibodies (**B**), or FITC-anti CD63 and PE-anti CD81 antibodies (**C**) for 2 h. After washing the sEVs via SEC qEV35, the sEVs were detected using NanoFCM.

It should be noted that the TIM4-affinity method can capture only PS-exposed sEVs, which may bias the results toward PS^+^-sEVs. We analyzed the characteristics of PS^+^-sEVs and PS^−^-sEVs using sEV flow cytometry to determine the differences between the subpopulations. NTA showed that PS^−^-sEVs accounted for 6.04% of the total sEVs in 293T cells. The expression ratios of the three EV markers, CD9, CD63, and CD81, were almost the same for all sEVs and PS^+^-sEVs; the ratio of single-positive sEVs for each marker tended to be slightly higher for PS^−^-sEVs (Fig. 4). These data indicate that the sEVs recovered by the TIM4-affinity method represent a major proportion of the sEV population and reflect almost the same sEV markers as whole sEVs. In summary, flow cytometry analysis of sEVs requires the use of highly pure sEV purification methods, such as the SEC and TIM4-affinity methods, for effective antibody washing.

### Flow cytometry of sEVs reveals changes in the sEV subpopulations

**Figure 5:**
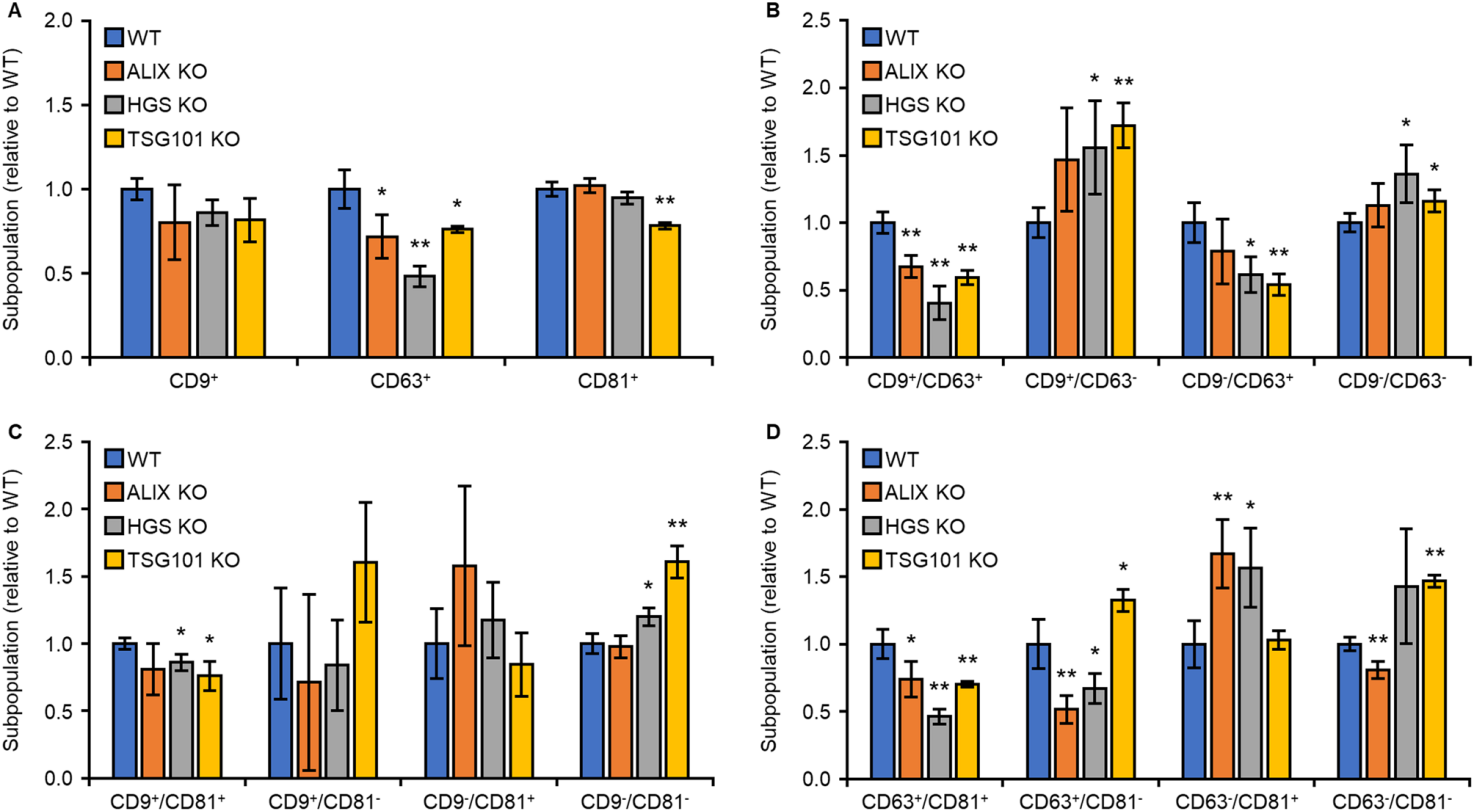
Analysis of sEV heterogeneity depending on sEV biogenesis pathways. 293T WT, ALIX KO, HGS KO, or TSG101 KO cells were cultured in Advanced DMEM-2% sEV-depleted FBS for 48 h. The sEVs contained in 10 K sup were captured via TIM4-affinity beads and stained with FITC-anti CD9 and PE-anti CD63 antibodies, FITC-anti CD9 and PE-anti CD81 antibodies, or FITC-anti CD63 and PE-anti CD81 antibodies for 2 h. After washing the sEV-bound TIM4-beads, the sEVs were released from the beads and detected using NanoFCM. Ratios of sEV subpopulations are presented. Data show mean ± SD. *P < 0.05, **P < 0.01, paired *t* test vs WT, *n* = 4. Flow cytometry data are presented in Supplementary Fig. 8.

We analyzed the sEV subpopulations generated by ESCRT molecules using sEV flow cytometry. The sEVs secreted from ALIX, HGS, or TSG101 KO 293T cells were captured using TIM4-affinity beads and stained with anti-CD9, anti-CD63, and anti-CD81 antibodies. In the analysis of WT-sEVs, CD9^+^/CD63^–^-sEVs were present in 17.4% of all sEVs, while sEVs positive for other single markers were rare (accounting for 3–5%) (Supplementary Fig. 8). Among the WT-sEVs, 30.7% were CD9^+^/CD63^+^-sEVs, 50.9% were CD9^+^/CD81^+^-sEVs, and 48.0% were CD63^+^/CD81^+^-sEVs, indicating that many WT-sEVs express multiple sEV markers (Supplementary Fig. 8). Depletion of ALIX resulted in no change in the percentage of CD9^+^- or CD81^+^-sEVs but resulted in a 28.2% decrease in CD63^+^-sEVs (Fig. 5A and Supplementary Fig. 8). Double staining for CD63 and CD81 showed depletion of ALIX reduced CD63^+^/CD81^+^-sEVs and correspondingly increased CD63^–^/CD81^+^-sEVs (Fig. 5D and Supplementary Fig. 8), suggesting that ALIX is involved in sorting of CD63 into sEVs without affecting the expression of CD81. A similar trend was seen in the results of CD63 and CD9 staining (Fig. 5B and Supplementary Fig. 8), suggesting that ALIX is involved in the sorting of CD63 into various sEVs. Depletion of HGS resulted in a 51.7% decrease in CD63^+^-sEVs (Fig. 5A and Supplementary Fig. 8), which was caused by the reduction in the levels of both CD9^+^/CD63^+^- and CD63^+^/CD81^+^-sEVs (Fig. 5B, D and Supplementary Fig. 8), suggesting that HGS is involved in the sorting of CD63 into a wide variety of sEVs. Depletion of TSG101 decreased the levels of CD63^+^- and CD81^+^-sEVs (Fig. 5A and Supplementary Fig. 8); furthermore, it decreased the number of double-positive sEVs and increased the number of double-negative sEVs (Fig. 5B–D and Supplementary Fig. 8). These findings suggest that TSG101 is involved in the biogenesis of a variety of sEVs, and HGS and ALIX contribute to the generation of a limited sEV subpopulation. Flow cytometry of sEVs clearly revealed changes in the levels of sEV subpopulations and is useful in unveiling sEV biogenesis and heterogeneity.

### Flow cytometry of sEVs is useful for detecting a disease-related molecule on sEVs

**Figure 6:**
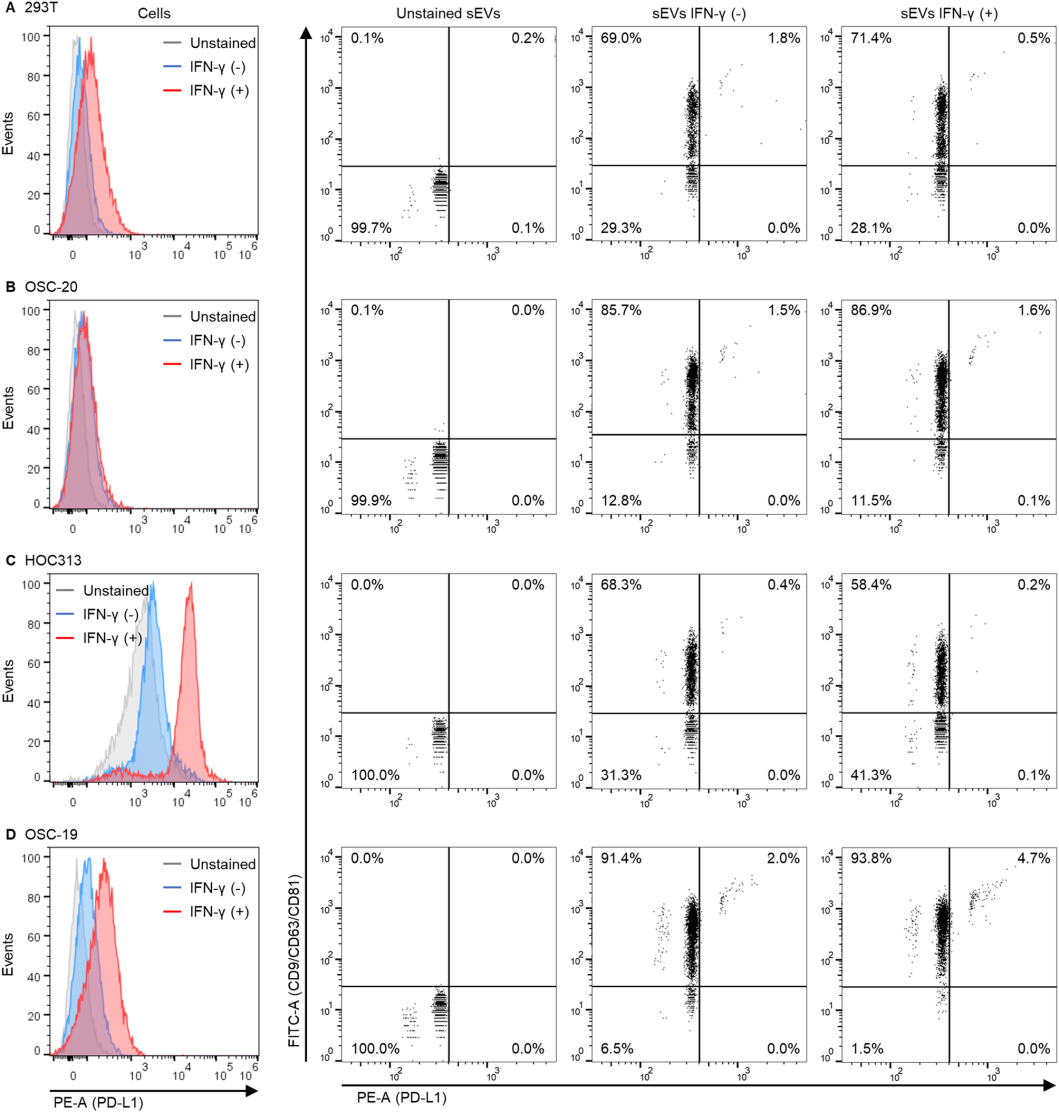
Analysis of cellular PD-L1 and sEV PD-L1 in different squamous cell carcinomas. 293T (**A**), OSC-20 (**B**), HOC313 (**C**), or OSC-19 cells (**D**) were cultured in Advanced DMEM-2% sEV-depleted FBS containing 0 or 100 ng/mL IFN-γ for 24 h. The cells were stained with PE-anti CD274 antibody at 4 °C for 60 min and detected using a cell analyzer (SA3800). Histograms show the expression of cellular PD-L1. The sEVs were captured by TIM4-affinity beads and stained with mixed antibodies of sEV markers (FITC-anti CD9, FITC-anti CD63, FITC-anti CD81), and PE-anti CD274 antibody for 2 h. After washing the sEV-bound TIM4-beads, the sEVs were released from the beads and detected using NanoFCM. Dot plots show the expression of sEV PD-L1.

The interaction between PD-1 expressed in T cells and PD-L1 expressed in cancer cells plays an important role in cancer immune checkpoints that suppress T-cell activation. Antibodies that inhibit this interaction are used in cancer immunotherapy as immune checkpoint inhibitors. It was recently reported that not only cellular PD-L1 but also sEV PD-L1 can suppress T cells^20,21^. Therefore, PD-L1-expressing sEVs secreted by cancer cells have attracted attention as therapeutic or diagnostic targets. However, the positive rate or expression level of PD-L1^+^-sEVs secreted by different cancer cells is not well understood because a single sEV analysis system has not yet been developed. Here, cellular PD-L1 and sEV PD-L1 expression was evaluated in three different cell lines of human head and neck squamous cell carcinoma and 293T cells (as non-cancer cells). The cells were stained with a PE-conjugated PD-L1 antibody and shown in histograms, and the sEVs were stained with a mixture of three FITC-conjugated antibodies against sEV markers and a PE-conjugated PD-L1 antibody and shown in dot plots. In 293T cells, the expression of cellular PD-L1 and the detection rate of PD-L1^+^-sEVs were low (Fig. 6A). Similar results were observed in OSC-20 cells (Fig. 6B). HOC313 cells stimulated with IFN-γ expressed high levels of cellular PD-L1, whereas the population of PD-L1^+^-sEVs was low (Fig. 6C). OSC-19 cells stimulated with IFN-γ expressed cellular PD-L1 at a moderate level, whereas the population of PD-L1^+^-sEVs increased from 2.0 to 4.7% depending on IFN-γ stimulation (Fig. 6D). These results indicate that there was no correlation between the expression levels of cellular PD-L1 and detection rates of PD-L1^+^-sEVs. Additionally, we found that PD-L1 was expressed in a limited population of sEVs (Fig. 6D), implying that PD-L1 may be sorted via a limited sEV biogenesis pathway. The staining method using mixed antibodies against sEV markers and an antibody against disease-related molecules allowed for the analysis of the heterogeneity of sEVs secreted from disease model cells. Single sEV analysis using flow cytometry is expected to be useful not only for elucidating the function of sEVs in disease but also for the diagnosis of sEV-related diseases.

## Discussion

In this study, we found that isolation of sEVs using ultracentrifugation was acceptable for detecting sEVs expressing fluorescent proteins. However, to detect sEVs stained with fluorescence-conjugated antibodies, a highly pure sEV isolation method, such as SEC or TIM4-affinity purification, is necessary to effectively remove unbound antibodies. AFM provided useful information regarding the purity of sEVs. It clearly showed that the sEVs isolated using TIM4-affinity purification contained few protein aggregates, while the ones isolated via SEC contained small-sized protein aggregates, which are acceptable for the flow cytometry analysis. Conversely, the sEVs isolated by ultracentrifugation contained a large number of protein aggregates of over 10 nm in size, which caused noise in the flow cytometry analysis. The sEVs isolated using density gradient method did not sink in a 1.08 g/mL solution, making their immobilization difficult on a mica substrate for AFM observation. The ultracentrifugation failed to separate sEVs from antibodies in most cases of antibody staining, except for single staining with anti CD63 antibody (Figs. 1 and 3 and Supplementary Figs. 4 and 5). It is affected by antibody aggregation, which limits antibodies that can be used. The TIM4-affinity method can recover sEVs of any size, resulting in the recovery of sEVs smaller than those recovered by qEV35. However, the TIM4-affinity method is limited to recovering PS^+^-sEVs. Consistent with our data, it has been reported that approximately 90% of sEVs expose PS on their surface in B16BL6 cells^22^; thus, the majority of sEVs can be recovered using the TIM4-affinity method. The sEV recovered by the TIM4-affinity method showed a slightly lower positive rate for sEV markers than those recovered by qEV35. There are two possible explanations for this low positivity rate. First, the antigens contained in PS^+^-sEVs may be too few to be detected. Because the surface area is proportional to the square of the particle size, a small decrease in particle size can result in a large decrease in the antigen content of the sEV. In fact, data plotted by fluorescent intensity and SS-H showed a trend toward smaller particle sizes with decreased fluorescence signals (Fig. 1). Second, aggregates of antibodies may not be completely removed in SEC, resulting in higher positivity rates. We found a number of events with very strong signals in the double-positive region not only in WT-sEVs of SEC samples but also in CD63 KO-sEVs (Fig. 3B). The same populations were observed in the ultracentrifugation samples (Fig. 3A) but not in the TIM4-affinity samples (Fig. 3C). We considered that these may be antibody aggregates similar in size to sEVs and are difficult to completely remove by SEC. Contrary to our expectations, density gradient centrifugation with iodixanol failed to isolate the sEVs from the unbound antibodies. Density gradient centrifugation is considered useful for separating vesicles with different densities but not for separating vesicles from soluble proteins, including antibodies.

Analysis of the sEV biogenesis pathway using flow cytometry revealed that depletion of ALIX decreased the levels of CD63^+^-sEVs but did not alter the levels of CD9^+^- or CD81^+^-sEVs. This is consistent with previous reports that ALIX is involved in the generation of CD63^+^-sEVs via the syntenin-syndecan pathway^3^. Considering a 28% decrease in CD63^+^-sEV levels from ALIX KO cells, the syntenin-syndecan pathway contributes to approximately 30% of the total sEV production in 293T cells. Depletion of HGS decreased CD63^+^/CD81^+^-sEV and CD9^+^/CD63^+^-sEV levels. HGS may be involved in more pathways in addition to the syntenin-syndecan pathway. Depletion of TSG101 reduced the levels of a variety of sEV subpopulations, suggesting that TSG101 plays an important role in many pathways, including the ESCRT pathway^2^. Analysis of the sEV biogenesis pathway using flow cytometry enabled the dynamic characterization of changes in the sEV subpopulation. Further studies using sEV flow cytometry could reveal how multiple sEV biogenesis pathways lead to sEV heterogeneity These studies provide useful information for developing therapies for sEV-related diseases.

PD-L1-expressing sEVs can reportedly promote cancer progression by suppressing patient immunity in vitro and in vivo^20,21^, and suppressing the secretion of PD-L1-expressing sEVs from cancer cells restores immunity and prolongs survival^21^. Consistent with the results of previous reports^21^, our data showed that cellular PD-L1 expression was low in OSC-20 cells, moderate in OSC-19 cells, and high in HOC313 cells, while sEV PD-L1 expression was positive only in OSC-19-sEVs. Secreting only few PD-L1^+^-sEVs from HOC313 cells may increase the expression of cellular PD-L1. Furthermore, PD-L1^+^-sEVs were limited to a subpopulation of OSC-19-sEVs. This suggests that the sorting of PD-L1 into sEVs may be mediated by a limited pathway for sEV biogenesis. Furthermore, Rab27a, nSMase2, and ALIX are involved in the secretion of PD-L1-sEVs^21,23^, although the activity of the molecules may differ between HOC313 and OSC-19 cells. The mechanism by which PD-L1 is sorted into sEVs is not fully understood. Elucidating the mechanism and its regulation would greatly facilitate cancer immune therapy.

Cells secrete a heterogeneous population of sEVs with various functions via different sEV biogenesis pathways. In the treatment of sEV-related diseases, it is important to elucidate which pathways generate disease-related sEVs, because the specific regulation of sEVs could promote treatment efficacy and reduce side effects. We demonstrated that highly pure sEV washing methods enabled the accurate single-particle analysis of sEVs, which can be used to analyze sEV biogenesis pathways, detect disease-related sEVs, and, in combination, elucidate the biogenesis pathways of disease-related sEVs, thereby enabling the development of therapeutic strategies for sEV-related diseases. Our findings can contribute to optimizing modern laboratory procedures for sEV detection, accelerating research of the mechanisms of sEV biogenesis, elucidating sEV-related disease mechanisms, and developing relevant diagnostic methods.

This study showed that the highly pure isolation methods were useful for flow cytometry of sEVs, but did not limit the use of ultracentrifugation, because it worked well in the staining with the antibody for human CD63 (Supplementary Fig. 3). We recommend comparison between wild-type and target molecule-deficient sEVs to develop flow cytometry of sEVs. Additionally, we consider that the best method for flow cytometry of sEVs has not yet been established because the SEC and TIM4-affinity methods have their own advantages and disadvantages (Table 1). It is important to choose a method that suits the purpose of individual studies.

**Table 1:** Characteristics of sEV washing methods.

sEV washing method	Characteristics	Effect	Advantages/Disadvantages
Ultracentrifugation	The standard method for sEV isolation. It should not be used for removing antibodies, but can be used for concentrating sEVs before staining	Low	Washing away of antibodies is insufficient
			In some cases, it increases nonspecific binding of an antibody to sEVs
Density gradient centrifugation	Separates sEVs from contaminants by centrifugation in different density solutions of iodixanol or sucrose	Low	Separating sEVs from antibodies is insufficient
SEC (qEV35)	Separates sEVs depending on their size	High	Surface molecules on sEVs do not affect isolation
			Concentrating sEVs is necessary because of dilution in the washing process
			Column is expensive
			Not recommended for serum analysis because of lipoprotein contamination^24^
TIM4-affinity method	Captures sEVs by TIM4 in the presence of Ca^2+^ and releases sEVs in the presence of EDTA	High	PS^–^-sEVs present at 5–10% are not recovered
			The process of isolation, staining, and washing can be performed using the beads
			Concentrating sEVs is very easy
			Many samples can be processed at once
			It requires small amounts of beads and is inexpensive

### Supplementary Information

Supplementary Information 1.

Supplementary Information 2.

## Data Availability

All data supporting the findings of this study are available within the paper and its supplementary information files or from the corresponding author upon reasonable request.
