# Supplementary material for: Precise analysis of single small extracellular vesicles using flow cytometry
Source: Sci Rep. 2024 Mar 29;14:7465. doi: 10.1038/s41598-024-57974-3 (PMC10980769; doi:10.1038/s41598-024-57974-3)
Supplement: Supplementary file 2 — Supplementary Information 2. [file 41598_2024_57974_MOESM2_ESM.docx]

**Supplementary information**

**Precise analysis of** **single small extracellular vesicles using flow cytometry**

Hisano Kobayashi^#,1,2^, Takayuki Shiba^#,1,4^, Takeshi Yoshida*^,1,3^, Dilireba Bolidong^1,3^, Koroku Kato^2^, Yoshiki Sato^5^, Mao Mochizuki^5^, Takafumi Seto^4^, Shuichi Kawashiri^2^, Rikinari Hanayama*^,1,3^

^1^Department of Immunology, Graduate School of Medical Sciences, Kanazawa University, Kanazawa, Ishikawa, Japan

^2^Oral and Maxillofacial Surgery, Graduate School of Medical Sciences, Kanazawa University, Kanazawa, Ishikawa, Japan

^3^WPI Nano Life Science Institute (NanoLSI), Kanazawa University, Kanazawa, Ishikawa, Japan

^4^Faculty of Frontier Engineering, Institute of Science and Engineering, Kanazawa University, Kanazawa, Ishikawa, Japan

^5^Meiwafosis Co. LTD, Tokyo, Japan

^#^These authors contributed equally to this research.

*Correspondence: Takeshi Yoshida and Rikinari Hanayama

(email: t-yoshida@med.kanazawa-u.ac.jp, telephone: +81-76-234-4563 and email: rikinari-hanayama@umin.ac.jp, telephone: +81-76-265-2725.

**
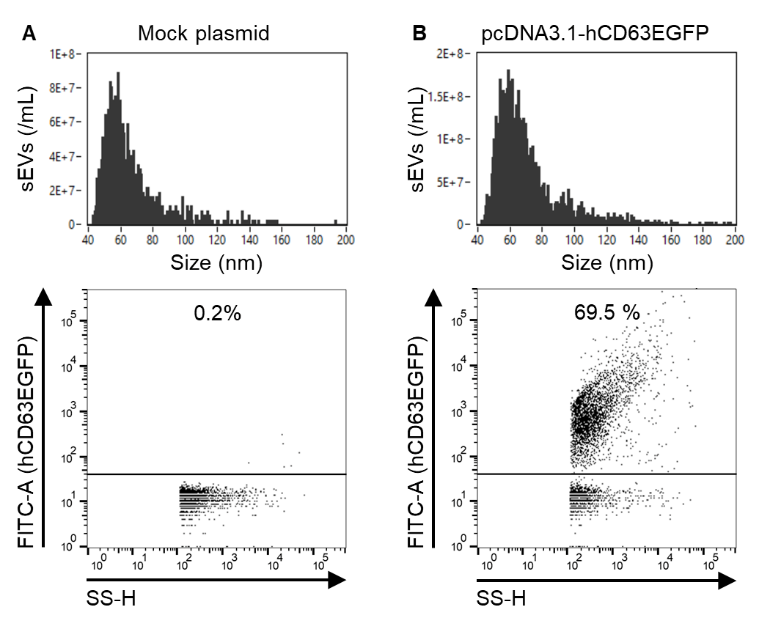
**

**Supplementary Figure 1 Detecting sEVs expressing a fluorescent protein by a sEV flow cytometer.** 293T cells transfected with the pcDNA3.1-hCD63EGFP plasmid or mock plasmid were cultured in Advanced DMEM-2% sEV-depleted FBS for 48 h. The 10 K sup was recovered from the cultured supernatant by serial centrifugation at 300 × *g* for 5 min, 2000 × *g* for 20 min, and 10 000 × *g* for 30 min. UC sEVs were recovered from the 10 K sup by ultracentrifugation at 100 000 × *g* for 2 h. SS and EGFP intensity of UC mock-sEVs (**A**) and UC hCD63EGFP-sEVs (**B**) were detected using NanoFCM. Particle size was calculated from the SS-H value using standard size beads.


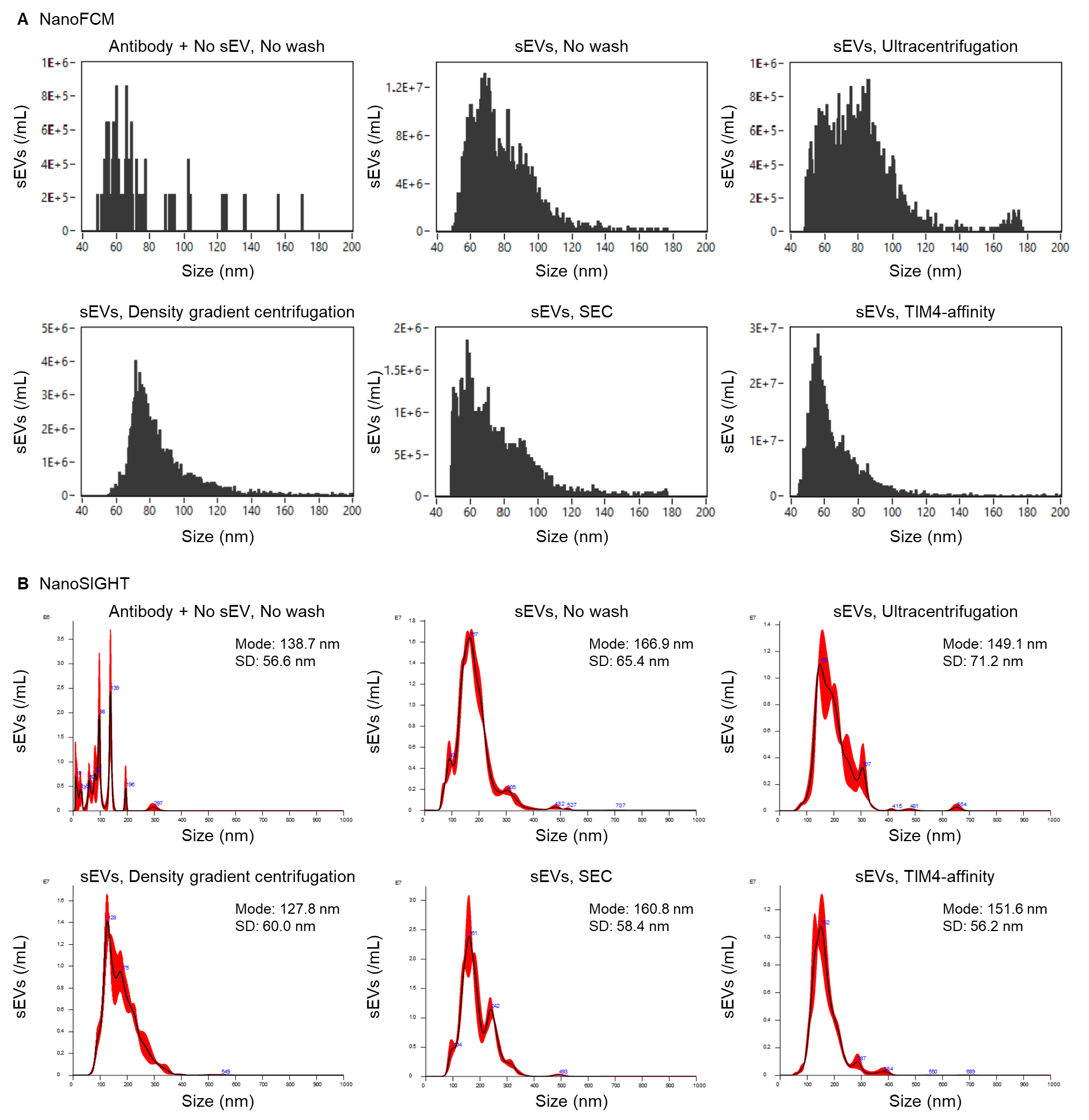


**Supplementary Figure 2 Sizes of sEVs isolated by different methods.** Particle sizes of no sEV with antibody and unstained WT sEVs washed via different methods were determined by using NanoFCM or NanoSIGHT. (**A**) SS intensity of each sample was detected using NanoFCM in Figure 1. Particle size was calculated from the SS-H value using standard size beads. (**B**) Brownian motion of sEVs in each sample was tracked using NanoSIGHT to calculate particle size.


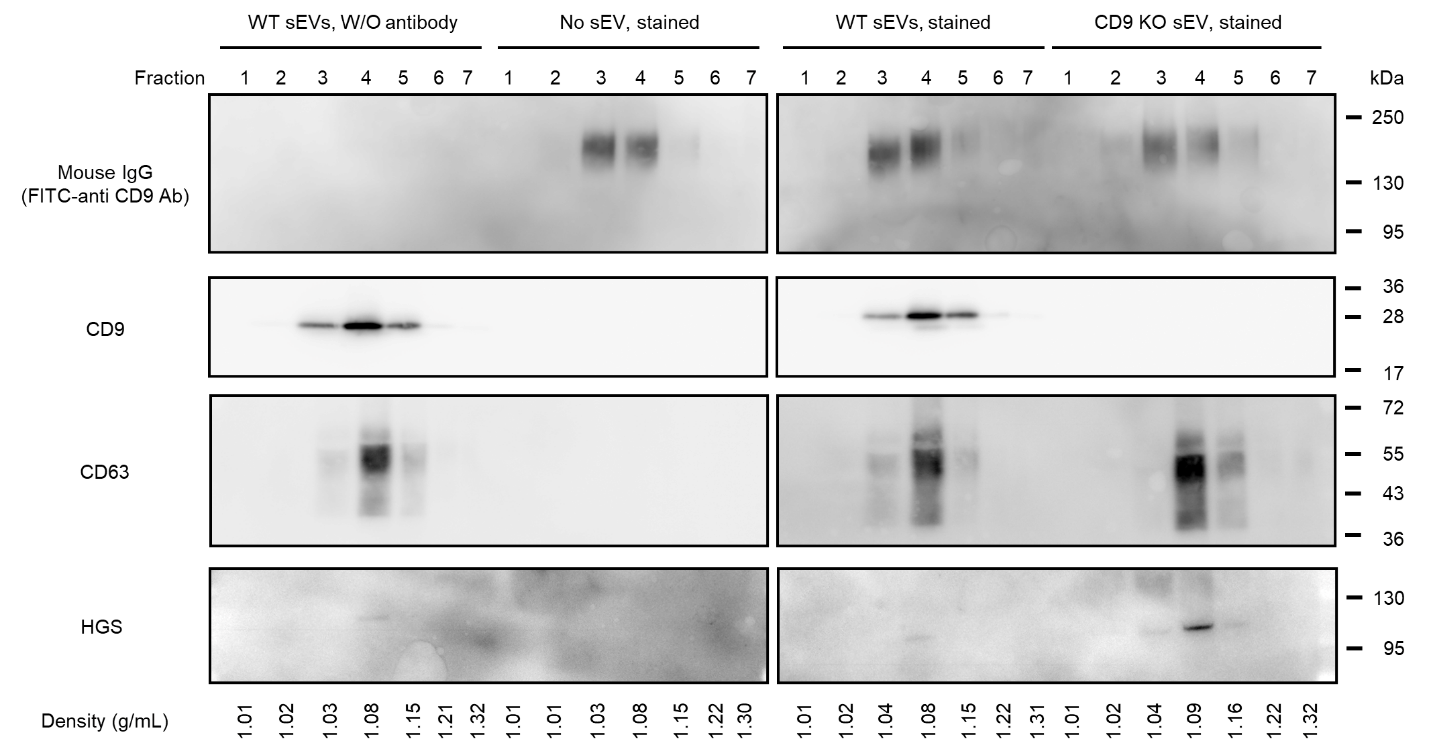


**Supplementary Figure 3 Distribution of antibodies and sEVs in density gradient centrifugation.** UC sEVs from 293T WT or CD9 KO cells were stained with FITC anti-human CD9 antibody for 2 h. The stained sEVs were placed at the top of the gradient density fractions and ultracentrifuged at 100 000 × g for 16 h. Fractions were carefully collected and separated in 5¬20 % SDS-PAGE under nonreducing condition. The FITC anti-human CD9 antibody was detected by HRP-conjugated anti mouse IgG antibody. The sEVs were detected by biotin-conjugated anti human CD9 antibody, biotin-conjugated anti human CD63 antibody or anti-HGS antibody for primary antibody and HRP-conjugated streptavidin or HRP-conjugated anti rabbit IgG antibody for secondary antibody.

**
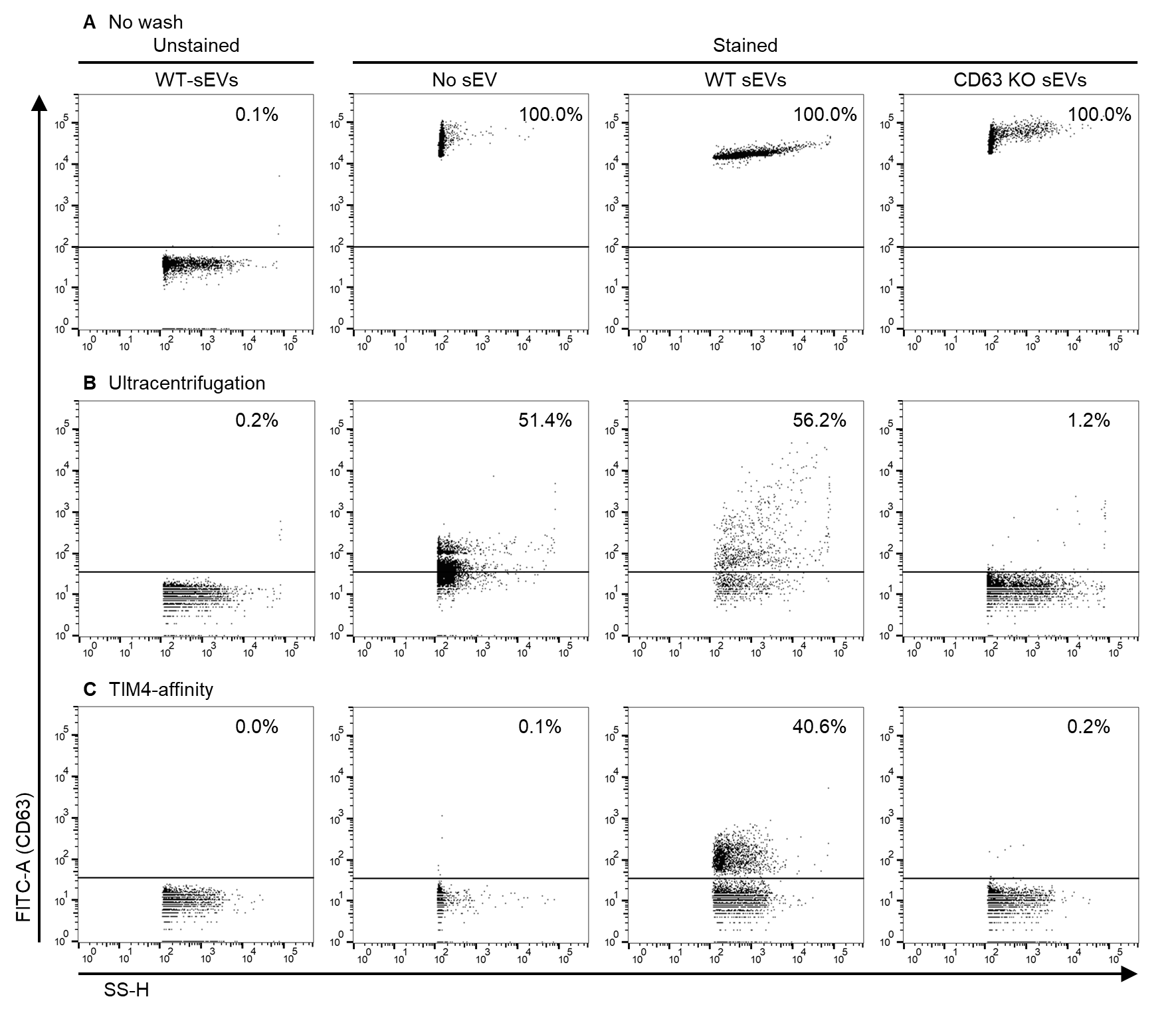
**

**Supplementary Figure 4 Evaluation of different washing methods for sEVs stained with anti-CD63 antibody.** UC sEVs from 293T WT or CD63­ KO cells were stained with FITC anti-human CD63 antibody for 2 h. The stained sEVs were not washed (**A**) or washed via ultracentrifugation (**B**) or the TIM4-affinity method (**C**). SS and FITC intensity of the sEVs were detected using NanoFCM.


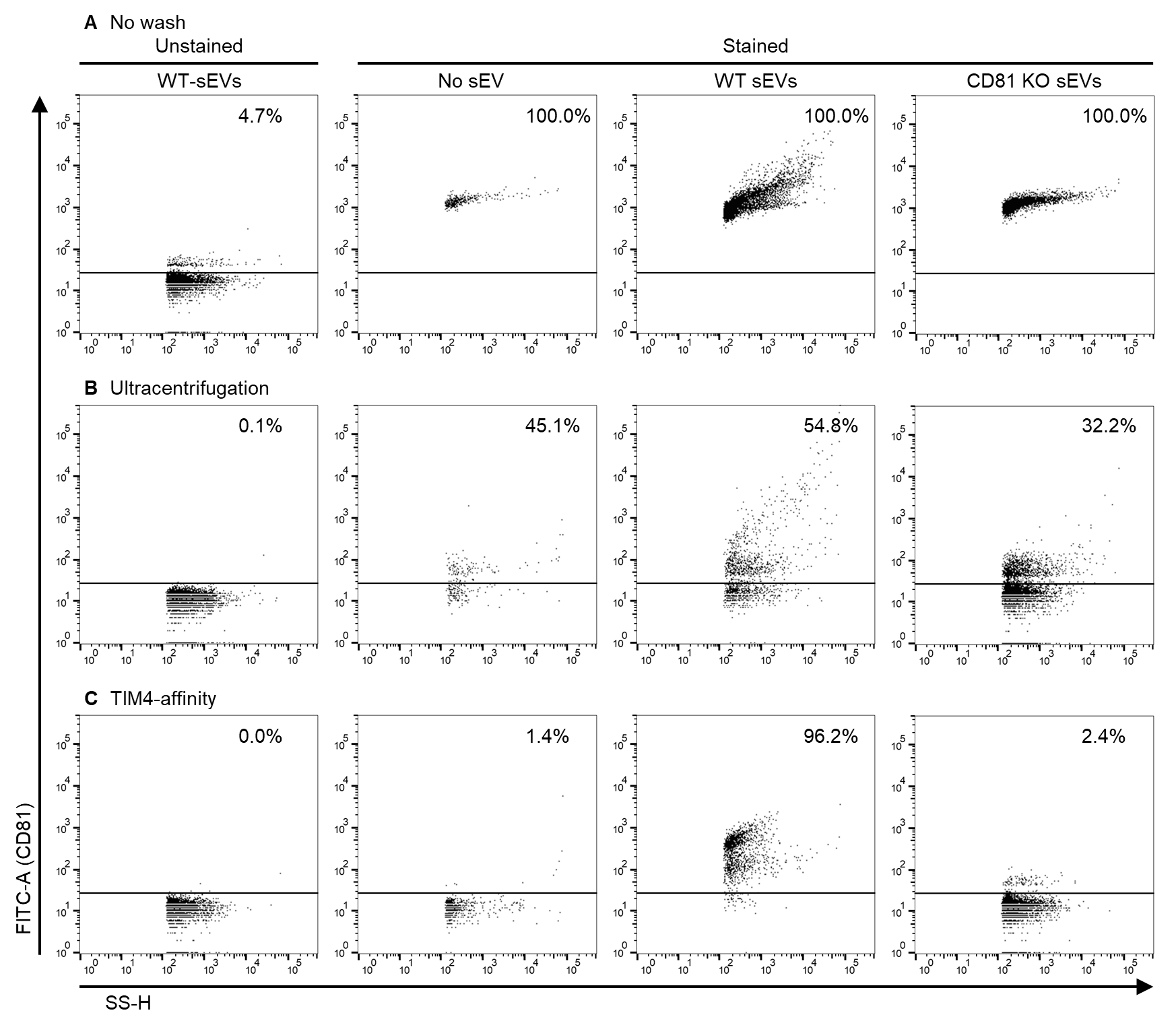


**Supplementary Figure 5 Evaluation of different washing methods for sEVs stained with anti-CD81 antibody.** UC sEVs from 293T WT or CD81 KO cells were stained with FITC anti-human CD81 antibody for 2 h. The stained sEVs were not washed (**A**) or washed via ultracentrifugation (**B**) or the TIM4-affinity method (**C**). SS and FITC intensity of the sEVs were detected using NanoFCM.

**
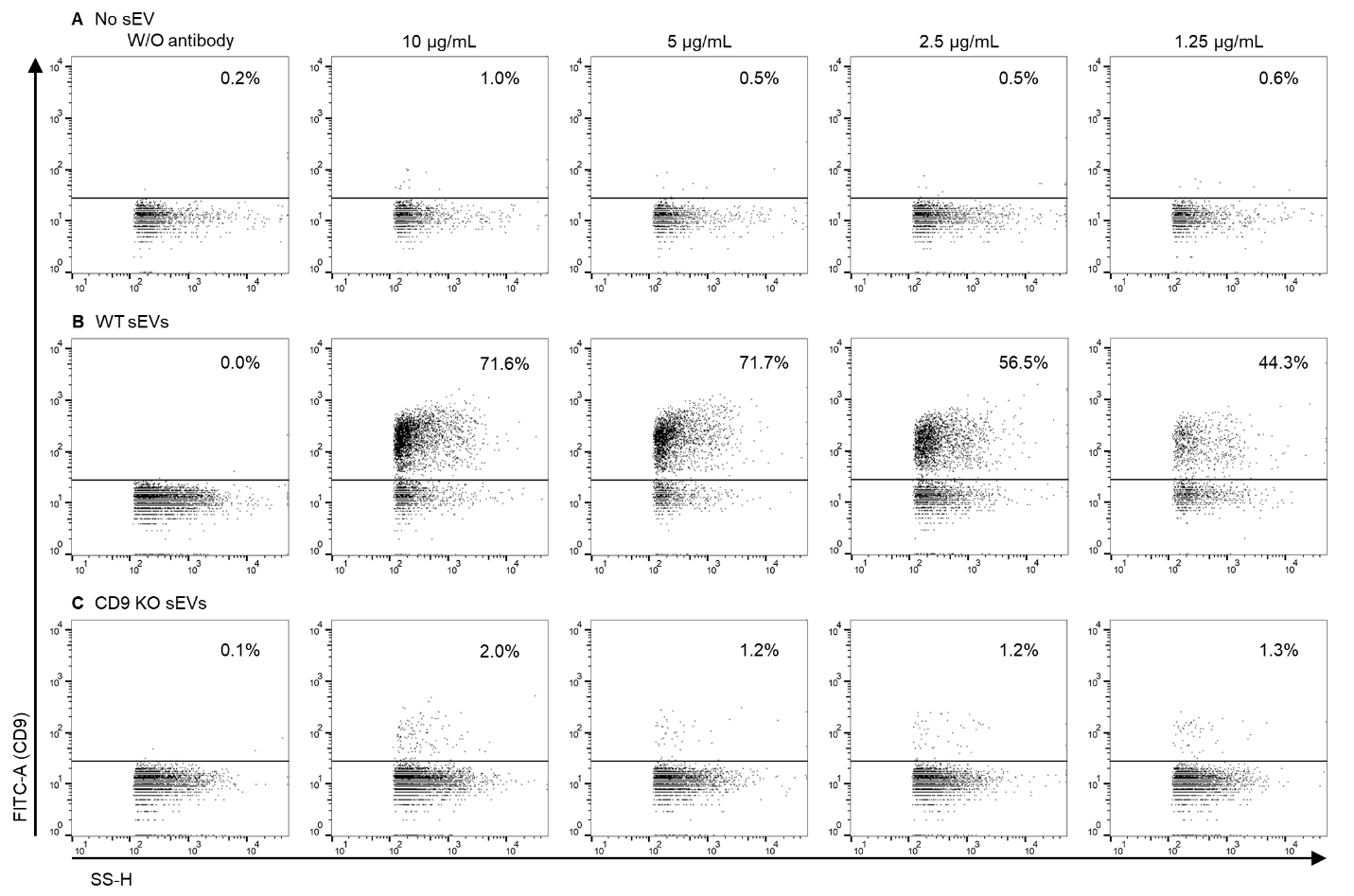
Supplementary Figure 6 Testing concentration of anti-CD9 antibody in TIM4-affinity method.** sEVs contained in 10K sup from 293T WT or CD9 KO cells were captured by TIM4-beads and stained with 0–10 µg/mL FITC anti-human CD9 antibody for 2 h. After the sEVs were detached from the beads, SS and FITC intensity of no sEV (**A**), WT-sEVs (**B**), or CD9 KO-sEVs (**C**) were detected using NanoFCM. Particle size was calculated from the SS-H value using standard size beads.


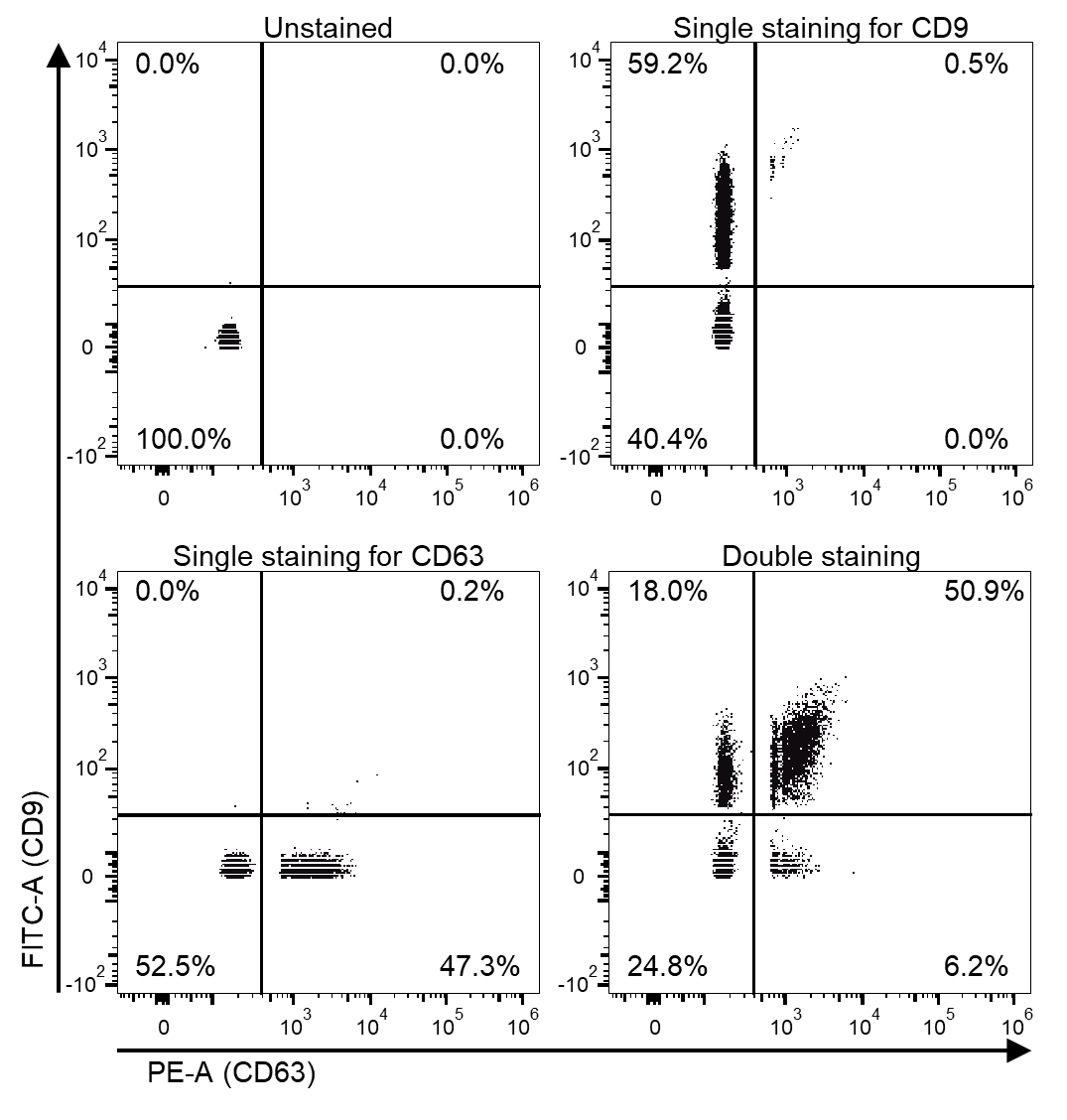


**Supplementary Figure 7 Single-stained controls for FITC-anti CD9 and PE-anti CD63 staining.** UC sEVs from 293T WT cells were stained with FITC anti-CD9 antibody, PE anti-CD63 antibody or both for 2 h. The sEVs were washed via the TIM4-affinity method. SS, FITC, and PE intensity of the sEVs were detected using NanoFCM.

**
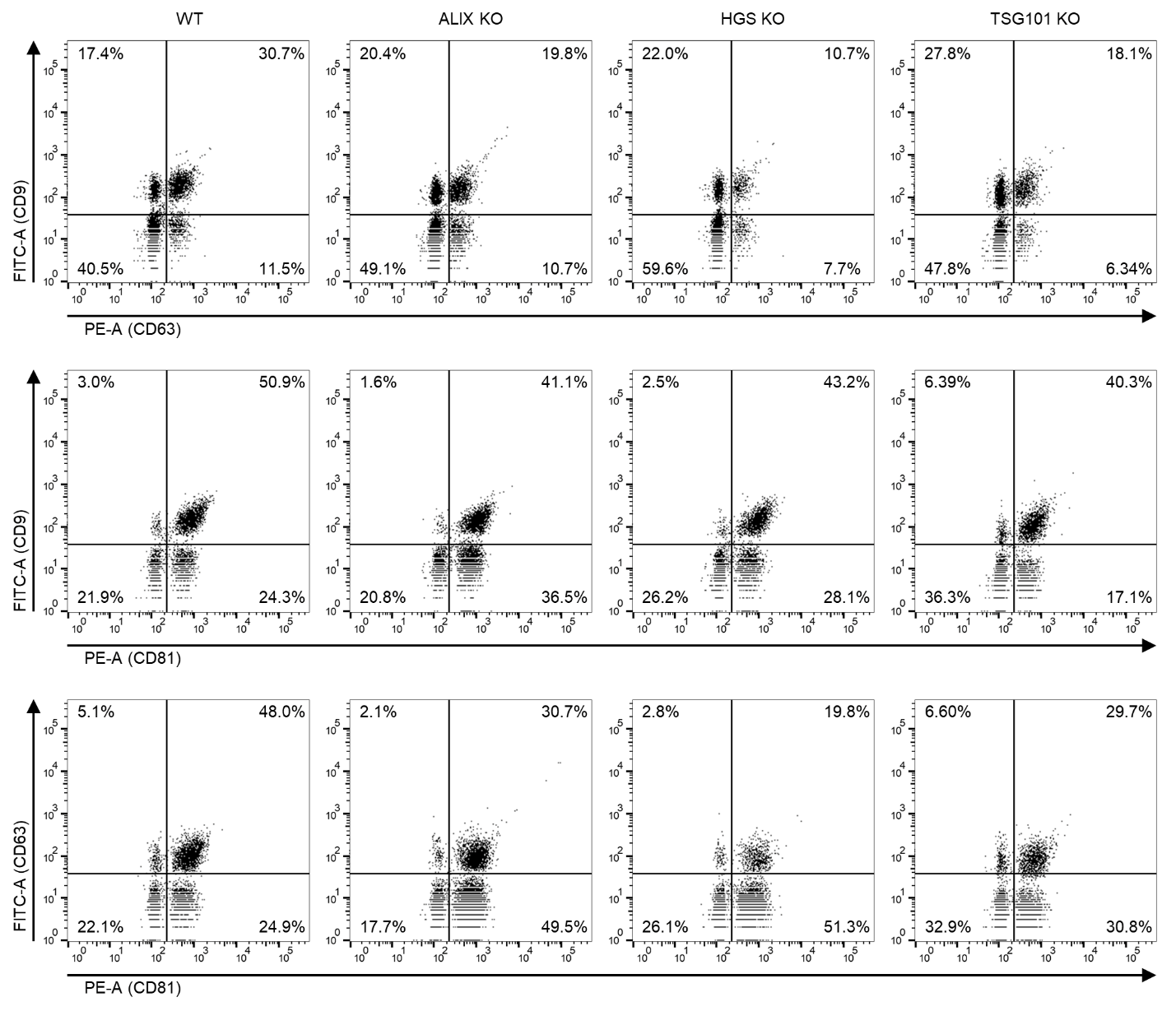
**

**Supplementary Figure 8 Analysis of sEV heterogeneity depending on sEV biogenesis pathway.** sEVs contained in 10K sup from 293T WT, ALIX KO, HGS KO, or TSG101 KO cells were captured by TIM4-affinity beads and stained with FITC anti-CD9 and PE anti-CD63 antibodies, FITC anti-CD9 and PE anti-CD81 antibodies, or FITC anti-CD63 and PE anti-CD81 antibodies for 2 h. After washing the sEV-bound TIM4-beads, the sEVs were released from the beads and detected using NanoFCM.

**Supplementary Table 1.** List of antibodies.

| Antigen | Antibody, Clone, Manufacturer, catalog # | Dilution for cellular staining | Dilution for staining sEVs |
| --- | --- | --- | --- |
| *Flow cytometry* |  |  |  |
| Human CD9 | FITC anti-human CD9, HI9a, BioLegend, 312104 | × 200 | ×10 |
| Human CD9 | PE anti-human CD9, HI9a, BioLegend, 312106 | - | ×10 |
| Human CD63 | FITC anti-human CD63, H5C6, BioLegend, 353006 | × 200 | ×10 |
| Human CD63 | PE anti-human CD63, H5C6, BioLegend, 353004 | - | ×10 |
| Human CD81 | FITC anti-human CD81, 5A6, BioLegend, 349504 | × 200 | ×10 |
| Human CD81 | PE anti-human CD81, 5A6, BioLegend, 349506 | - | ×10 |
| Human PD-L1 | PE anti-human CD274, 29E.2A3, BioLegend, 329706 | × 200 | ×10 |
| *Western blot* |  |  |  |
| Human HGS | HGS antibody (C2C3), C-term, GeneTex, GTX101718 | ×1000 | ×1000 |
| Human ALIX | Alix Mouse mAb, 3A9, Cell Signaling, 2171 | ×1000 | - |
| Human TSG101 | Anti-TSG101 antibody, GeneTex, GTX118736 | ×1000 | - |
| Mouse IgG | HRP-conjugated anti mouse IgG antibody, BioLegend, 405306 | - | ×3000 |
| Human CD9 | biotin-conjugated anti human CD9 antibody, BioLegend, 312112 | - | ×1000 |
| Human CD63 | biotin-conjugated anti human CD63 antibody, BioLegend, 353017 | - | ×1000 |
| Biotin | HRP-conjugated streptavidin, BioLegend, 405210 | - | ×3000 |
| Rabbit IgG | HRP-conjugated anti rabbit IgG antibody, BioLegend, 406401 | - | ×3000 |

**Full-length blots**

Supplementary Figure 3

**
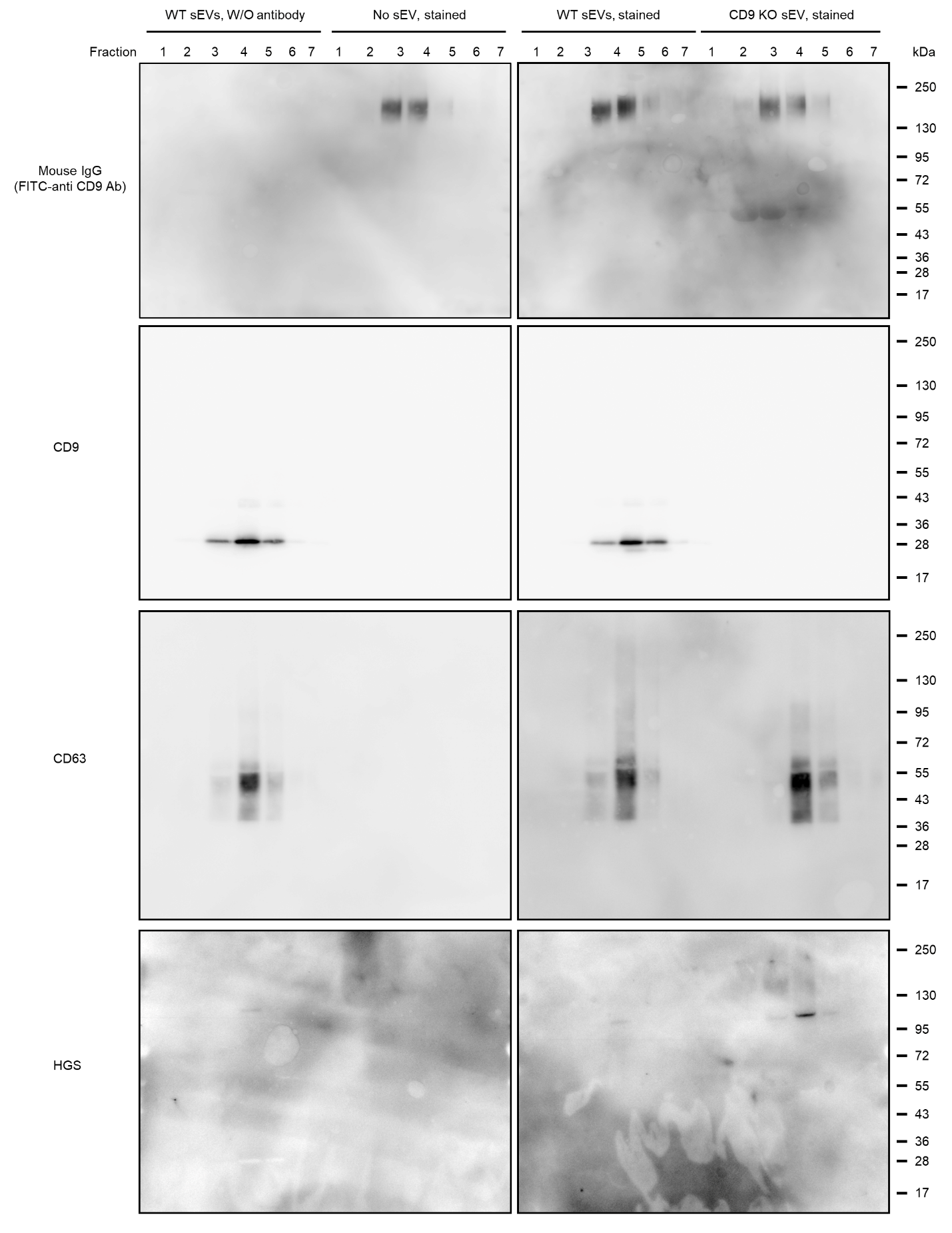
**
